# Supplementary material for: Colchicine reduces lung injury in experimental acute respiratory distress syndrome
Source: PLoS One. 2020 Dec 2;15(12):e0242318. doi: 10.1371/journal.pone.0242318 (PMC7710059; doi:10.1371/journal.pone.0242318)
Supplement: S1 Table — (DOCX) [file pone.0242318.s001.docx]

**S1 Table. Monoclonal antibodies used for identification of leukocytes sub-populations**

| Antibody | Fluorochrome | Clone | Supplier | Dilution |
| --- | --- | --- | --- | --- |
| Live-Dead | 7AAD |  | Biolegend | 1/20 |
| CD32 | N/A |  | BD | 1/100 |
| CD45 | Alexa Fluor 700 | OX-1 | Biolegend | 1/100 |
| Granulocytes | FITC | HIS48 | BD Pharmingen | 1/200 |
| CD11b | V450 | WT.5 | BD Horizon | 1/100 |
| CD43 | PE | W3/13 | Biolegend | 1/200 |
| CD45R (B220) | BV711 | HIS24 | BD Horizon | 1/50 |
| CD3 | BV605 | 1F4 | BD Horizon | 1/50 |
| CD4 | APC-Cy7 | OX-35 | BD Pharmingen | 1/50 |
| CD8a | BV786 | OX-8 | BD Horizon | 1/50 |
| CD161 | Alexa Fluor 647 | 10/78 | BD Pharmingen | 1/400 |
