# Supplementary material for: Colchicine reduces lung injury in experimental acute respiratory distress syndrome
Source: PLoS One. 2020 Dec 2;15(12):e0242318. doi: 10.1371/journal.pone.0242318 (PMC7710059; doi:10.1371/journal.pone.0242318)
Supplement: S2 Table — (DOCX) [file pone.0242318.s002.docx]

**S2 Table. Primer sequences used for quantitative real-time PCR of rat target genes**.

| **Gene** | **Forward primer (5'-3')** | **Reverse primer (5'-3')** |
| --- | --- | --- |
| ACTB | CCCTAAGGCCAACCGTGAA | GAGGCATACAGGGACAACACAG |
| Arg1 | GCAGAGACCCAGAAGAATG | GCCAGAGATGCTTCCAAT |
| ATP1A1 | TGCTCTCTCCTCTTTCTA | CTTCTTGCTCTTCTTGTC |
| ATP1B1 | GCTAAACATCATCAGGTTCCT | TTGGGTTCACTGGGCATA |
| Calcrl | GTTCAGACATCCAGATAGTAA | GAGAGGCAATAGATAATCCAT |
| Casp1 | CGAGACCTGTGCGATCAT | GCTGATGGACCTGACTGAA |
| CD31 | TCATTGGAGTGGTCATTG | TGTTGGAGTTCAGAAGTG |
| CD68 | AAGCAGCACAGTGGACAT | TTGTATTTCCGCAACAGAAGC |
| CXCL1 | TCATAGCCACACTCAAGAA | GGACACCCTTTAGCATCT |
| CXCR2 | AAGCCTTGAGTCACAGAG | AATATCTCCACTGAAGAAGTCT |
| E-sel | GTCCAGTTGTAAGTTCTC | ACTCATGTTCATCTTTCC |
| GAPDH | GGCTGGCATTGCTCTCA | GTCCACCACCCTGTTGCTGTA |
| GPR84 | TCAGGTGAGTCTCCATCAT | AACAGGGTGAGCACATTG |
| ICAM-1 | TTGGAGACTAACTGGATGA | CTCTGGGAACGAATACAC |
| IL-1b | GACAGAACATAAGCCAACA | ACACAGGACAGGTATAGATT |
| IL-6 | TGAAGAACAACTTACAAGATAAC | CATTAGGAGAGCATTGGAA |
| IL10 | AGCAGGTGAAGAATGATT | GCAGTTGATGAAGATGTC |
| IL13 | CACAAGACCAGAAGACTT | GCCATTCAATATCCTCTG |
| IL33 | CACACTGAGTATCCAAGG | CGTAACATCCATTCTCCAA |
| MCP-1 | TCACCAGCAGCAGGTGTCC | CACAGATCTCTCTCTTGAGCTTGG |
| MMP2 | CACAACCAACTACGATGATGA | GCTGCCACAAGGAATAGG |
| MMP9 | CCGACTTATGTGGTCTTCC | CAGGTAATCCTCTGCCAG |
| NLRP3 | TAAGAAGGACCAGCCAGAG | CGAGATGCGGGAGAGATA |
| PAI-1 | GATGCTATGGGATTCAAT | GTACTGATCTCATTCTTGT |
| Ramp1 | TGTCAAAAGGGAAGATGGA | GTTGCTGTAATACCTGCTAAT |
| SCNN1A | ATCAACCTCAATTCAGACAAG | GCGAGTGTAGGAAGAGTT |
| SCNN1G | CTGTGATGCCAGGAACTTC | ATGGAGGTGCTGAGGATG |
| TIMP-1 | ACACGCTAGAGCAGATAC | GCTGGTATAAGGTGGTCTC |
| TNFa | GTGATCGGTCCCAACAAGGA | GATGAGAGGGAGCCCATTTG |
| VCAM-1 | GCCTCGCTAAGTTACACAG | AGCAGGTCAGGTTCACAG |
| XBP1 | AAGTGGTGGATTTGGAAGAAG | CCTTGGACTCTGCCTCTG |
